# Violet teaming as a resilient framework for bridging civilian-military electronic health record interoperability

**DOI:** 10.3389/fdgth.2026.1761714

**Published:** 2026-06-10

**Authors:** Beth Ellinport, Alexander Titus, Benjamin D. Trump

**Affiliations:** 1 Environmental Laboratory, US Army Engineer Research and Development Center, Concord, MA, United States; 2 In Vivo Group Seattle, Washington, DC, United States; 3 Tor Intelligence, LLC Apex, NC, United States

**Keywords:** cybersecurity, electronic health record, interoperability, military-Civilian, violet teaming

## Abstract

The rapid digitization of healthcare through electronic health records (EHRs) and artificial intelligence (AI) is transforming clinical decision-making, data integration, and healthcare delivery. However, increasing dependence on interconnected digital systems introduces significant cybersecurity, interoperability, ethical, and operational challenges that EHRs already face, further compounded by the operational complexity of military-civilian healthcare environments where information-sharing requirements are highly sensitive. AI-enabled systems intensify these concerns through opaque decision processes, extensive data demands, and expanding attack surfaces. This article introduces and applies an integrated cybersecurity framework that advances beyond traditional purple teaming by incorporating human factors, governance, ethical considerations, and operational continuity into resilience planning. The paper highlights how this approach can improve secure interoperability, identify systemic vulnerabilities earlier in the development and deployment lifecycle, and support more resilient military-civilian health data exchange architectures. The article further argues that resilience in digital healthcare systems cannot be achieved through technical safeguards alone but instead requires a sociotechnical framework that balances cybersecurity and clinical operations. Originally conceptualized in the context of the biotechnology governance, this paper extends violet teaming into the domain of military-civilian electronic health record interoperability through an illustrative vignette, demonstrating how the framework may support proactive resilience, governance, and cybersecurity integration within complex healthcare data ecosystems, ultimately mitigating risks to patient safety and human life.

## Introduction

Healthcare's rapid digitization, driven by the widespread adoption of electronic health records (EHRs) and artificial intelligence (AI), has introduced a paradox: these tools improve care delivery, but also expose patients, providers, and health systems to new risks. In information exchanges between military and civilian healthcare systems, classified exposures, incompatible infrastructure, and inconsistent access policies can create critical gaps in care and trust. As health IT systems integrate with AI, traditional cyber security strategies are insufficient for protecting the sensitive, high-stake health data. This paper applies violet teaming as a recent and novel cybersecurity framework designed to bridge these gaps.

Violet teaming builds upon existing cybersecurity methodologies, extending red and blue teaming by incorporating social and ethical dimensions of healthcare security (1). While many socio-technical frameworks articulate principles for trustworthy or ethical health IT, violet teaming is distinct as a method. An iterative, adversarial test-and-adapt cycle that operationalizes those principles inside cybersecurity practice. It extends purple teaming by adding sector impact testing and system & policy adaptation, making human trust, clinical usability, and ethical access decisions testable outcomes that directly drive technical and governance changes.

EHRs address long-standing challenges, including missing records, illegible notes, and inefficient coordination. Today, nearly all U.S. hospitals and office-based physicians use certified EHR systems (2). Yet these systems often overlook practical user needs, especially in high-risk settings, undermining system resilience during crises or transitions.

The stakes of poor system design are high. Healthcare organizations now experience some of the most frequent and costly cyberattacks, with data breaches averaging $10.93 million per incident, up nearly 30% since 2020 (3). Despite growing trust in digital healthcare systems, 66% of individuals express concerns about the safety of their personal medical data (4). Medical records can sell for thousands of dollars, far more than credit card data, and contain deeply personal, irreplaceable information (5). Breaches threaten not just finances, but patient safety, privacy and trust.

AI integration further complicates this landscape. While AI improves diagnostic accuracy, treatment planning, and operational efficiency, it introduces ethical concerns, biosecurity risks, and potential data misuse (6). AI algorithms require substantial patient information for training and decision-making, with the potential of introducing new points of vulnerability. AI-driven systems also function as “black boxes,” that obscure accountability, consent, and potential biases (7). Ensuring secure, responsible, and unbiased AI operations is an urgent policy and governance priority.

Current cybersecurity approaches may be insufficient to address vulnerabilities emerging from AI advancements. Tradition security measures focus on preventing unauthorized access without incorporating the complex, interconnected nature of modern health systems (8). With the AI-enablement of health IT, isolated, threat-specific defenses may become insufficient as systems grow more interconnected. A whole-of-system approach can ensure systems can respond, recover, and adapt—safeguarding patient care and continuity.

Exchange points between health systems can amplify vulnerabilities through variations in data governance policies, technical standards, and institutional priorities (8). AI-driven health data processing adds complexity and motivates the development of new methodologies to integrate technical and human-centered considerations within cybersecurity strategy (9). Current evaluations focus mostly on systems interactions while ignoring the people affected. Violet teaming provides a holistic and dynamic stress-test methodology to help identify hidden gaps and support real-world resilience.

These challenges become heighten at the intersection of military and civilian healthcare. The Military Health System (MHS) maintains highly sensitive health records including classified operational data, like chemical and biological exposures, combat injury reports, and deployment health assessments (10). When service members transition to civilian care, their data must be securely transferred across networks with differing cybersecurity standards and infrastructures. Although the Health Insurance Portability and Accountability Act (HIPAA) establish baseline security requirements, compliance alone has not entirely prevented breaches (11). Military-civilian data interoperability requires sharing information while protecting sensitive data and operational security.

Violet teaming addresses these challenges by testing systems under complex, human-centered scenarios. It assembles technical experts, clinicians, patients, and legal experts to simulate real-life security failures to identify causes. This paper details how violet teaming can strengthen interoperability, particularly in military-civilian health exchanges involving AI.

## The evolution of electronic health records

EHRs emerged in the 1960s to, originally, address the inefficiencies of traditional paper-based records. These EHRs were digital versions of paper charts used for diagnosis and treatment, but they rarely traveled outside a single practice. Later, EHRs expanded to patients' total health, enabling information sharing across providers (12). These systems looked to streamline workflows and improve storage, access, and sharing. In the 1970s–80s, a few institutions, like the VA, piloted digitized records (13, 14). Widespread adoption faced hurdles such as high costs, incompatible standards, and poor interoperability. In 2004, the Office of the National Coordinator for Health Information Technology (ONC), now known as ASTP, was created to accelerate the nationwide adoption and integration of health IT, particularly EHRs. In 2009, the Health Information Technology for Economic and Clinical Health (HITECH) Act offered financial incentives to healthcare providers (15, 16). By 2021, nearly 80% of office-based physicians and 96% of non-federal acute care hospitals in the United States had adopted certified EHR systems, an increase from just 28% of hospitals and 34% of physicians in 2011 (17).

Modern EHR systems give patients access to their health records, diagnoses and treatments, and allow healthcare providers to communicate through EHR portals, improving timely care (16, 18). For physicians, EHRs streamline charting, automate reminders, and support clinical decisions, improving workflow efficiency and reducing the likelihood of errors (18). However, widespread adoption brought significant cybersecurity challenges. In the 2015 Anthem Blue Cross breach, thieves stole 78.8 million patient records, while a breach in 2023 affected 8.9 million individuals (5). Cyberattacks on EHR systems can target sensitive personal health information (PHI) and lead to costly ransom demands. Such attacks galvanize stronger protections.

At the same time, interoperability barriers in EHR systems reflect two compounding but distinct challenges. In acute inpatient and ambulatory care, the market has become highly concentrated, with EPIC Systems and Oracle Health collectively controlling almost a 70 percent share of hospital systems and inpatient beds (19–21). Thus, the interoperability barriers for large health systems stem less from vendor diversity than from proprietary architectures and business incentives from dominant vendors whose platforms are inherently built to discourage cross-vendor data exchange (19). At the same time, specialty, behavioral health, post-acute, and smaller ambulatory practice settings remain served by genuinely fragmented EHR systems (22) many which also fail to exchange data seamlessly due to vendor restrictions and legacy platforms (23). This is especially pronounced when patients move between healthcare providers or when military-civilian systems exchange sensitive health information.

Efforts to standardize data exchange have been led by organizations such as Health Level 7 International (HL7). Founded in 1987, it developed messaging standards to ensure interoperability between different EHR systems (24). The HL7 messaging framework structures data in segments for demographics, labs, histories (25), although older versions of HL7 lack encryption. More modern standards, like Fast Healthcare Interoperability Resources (FHIR), use web technologies, APIs, for data exchange, and provide real-time access to patient records (26). Yet, FHIR implementations still embody server and access control vulnerabilities. Legacy systems running old protocols have vulnerabilities from unencrypted imaging data, while inconsistent security practices across providers worsens these gaps.

In addition, recent federal efforts, including the Trusted Exchange Framework and Common Agreement (TEFCA), seek to enable nationwide health information exchange through standardized networks (27). However, participation remains voluntary, and current requirements emphasize basic data exchange rather than full semantic and functional interoperability. This still allows for these dominant vendors such as Epic Systems to remain compliant while maintaining internal networks that enable more seamless data sharing within their own ecosystems, reinforcing existing lock-in dynamics (19).

Today, the healthcare industry continues to embrace digital solutions, and Artificial Intelligence (AI) and machine learning (ML) are providing new opportunities. In 2020, the $21.6 billion investments into digital health companies doubled the 2019 investments (28). These tools personalize care, enhance diagnostics, and detect unusual patterns to spot rare conditions or emerging diseases (9). They rely on massive amounts of sensitive data and complex algorithms that can misclassify, misinterpret, or be exploited if security is weak. Genetic data used for personalized treatment plans raises unique privacy risks if leaked or misused. These challenges transcend technical controls; they require engaging patients, clinicians, and communities to ensure AI-driven EHR systems balance security, transparency, and timely care. EHR resilience must integrate technical security, transparent AI governance, and ethical oversight. AI integration into military and civilian health data exchange holds significant promise for improving interoperability but also raises major concerns about data privacy and the handling of sensitive information. AI systems can help tag and share military-specific health data across disparate platforms, yet they may introduce new risks to the security and confidentiality of classified health records.

## The current landscape of EHRs: civilian vs. Military Systems

While both civilian and military EHR systems are built on like infrastructure, they face distinct core issues stemming from operational requirements, population needs, and regulatory frameworks, and these differences affect care delivery, data sharing, and overall system resilience. Military EHR systems serve a globally dispersed population operating in high-risk environments to provide both routine care and operational readiness. In contrast, civilian EHR systems primarily focus on consistent localized patient care. Systems like MHS Genesis aim to centralize and standardize health information across the Department of Defense, VA, and few civilian networks, but integration is still incomplete. This complicates care, particularly for service members whose health histories span both military and civilian settings.

## Civilian EHR systems

Although more than 700 government-certified EHR products exist, real-world interoperability barriers are better understood as the result of these dominant vendor ecosystems and misaligned data-sharing incentives, rather than vendor diversity alone (19, 29, 30). The ONC's core data requirements, United States Core Data for Interoperability (USCDI and USCDI +), aim to establish a consistent set of patient information—patient demographics, medication lists, allergies, lab results, diagnostic reports, and clinical notes—to support data exchange (31). However, records often remain scattered across systems, making it hard for providers to see a patient's full history and smaller clinics may lack advanced EHR capabilities altogether (23).

Initiatives like the 21st Century Cures Act push for open APIs and prevent “information blocking” (32). While regulations like HIPAA safeguard privacy but can encourage organizations to err on the side of caution to avoid breaches or penalties (33). The ONC's efforts to standardize essential EHR data, such as patient demographics, clinical notes, and lab results, are crucial steps toward solving these issues.

## Military EHR systems

The military healthcare system serves approximately 9.6 million active-duty personnel, retirees, and their families worldwide, providing care both within the military health infrastructure and through civilian healthcare providers (34). Unlike civilian EHRs, military records are operationally required to follow service members across combat zones, bases, VA hospitals, and civilian providers, including under conditions involving classified or operationally sensitive information. Historically, the DoD relied on multiple disconnected systems, creating fragmentation and inefficiency in sharing patient data (35, 36).

To solve this, the DoD launched MHS Genesis in 2017, built on the commercial Oracle Health platform aligning it with dominant civilian EHR systems, to consolidate legacy systems into a single, integrated platform that connects military, VA, and some civilian care. It assembles inpatient, outpatient, dental, and behavioral health records, and supports secure exchange through networks like eHealth Exchange. However, rollout has faced delays, cost overruns, and usability issues (37).

The most consequential gap emerges not solely from the ecosystems itself but from the absence of military specific clinical data in civilian care environments. Civilian EHRs rarely capture critical military-specific data like CBRN exposure, combat injuries or deployment health risks, information essential for treating PTSD, TBI, and toxic exposures (38, 39). Service members' combat zone exposures may cause symptoms that are difficult to diagnose without knowledge of their military background. Yet privacy laws like HIPAA, along with additional DoD regulations, can make sharing data cumbersome. The “Military Command Exception,” allows for the disclosure of certain health information to military command authorities under specific circumstances related to fitness and mission requirements, balancing operational readiness with privacy (40).

Improving EHR system operability and integration of military-specific health data, can ensure that military-affiliated patients receive high-quality, coordinated care anywhere they seek treatment.

## Gaming EHR: violet teaming

Traditional cybersecurity divides into offensive and defensive operations; Red teaming simulates attacks to find vulnerabilities, mimicking real-world threats, while blue teaming defends systems through monitoring, detection, and response. This relationship of attack and defense define two approaches to cybersecurity (41). Together, cybersecurity literature calls this feedback loop purple teaming, which involves collaboration between red team (attack) and blue team (defense) (42).

These feedback loops are often applied *post hoc*. Systems are typically assessed after deployment, once vulnerabilities emerge. This methodology focuses almost entirely on the technical impacts of a vulnerability and solution while ignoring human factors, patient trust, clinician usability, and ethical data handling.

A newer approach, violet teaming, emerged in 2023 to close this gap by integrating user perspectives directly into cybersecurity strategy (43). It retains red and blue team tactics but brings them into real-world contexts, examining how systems intersect with human behavior and policy constraints (1). This expands the traditional cycle to test social impact and sector-specific realities, moving cybersecurity from a reactive approach to a proactive resilience-building framework. In a violet teaming paradigm, the process may end up with more technical risk then a purple team would exercise because a perfect security posture limits a key stakeholder in a way that causes harm beyond the technical system. Although, originally developed in response to AI–biotech risks, violet teaming aligns naturally with healthcare, where technical reliability and human-centered decision-making must coexist (1).

Operationally, violet teaming is most effective when implemented upstream in the training and development stages of the product lifecycle. Organizations should integrate assessments at key intervention points, to monitor, keep track of, and understand decision pathways are exposed, un-tested, or entirely unexamined. Each cycle follows a similar approach where teams identify high-risk scenarios, such as military-civilian record transfer, and conduct collaborative simulations that stress-test both technical infrastructure and human workflows. It is important to note that purple teams do take stakeholder feedback into account, but everything is related to the technical system. Violet teams make decisions that may not actually be directly related to a technical-only lens, but one that is primarily driven by societal impact. The object is to develop systems as a sociotechnical entity, rather than purely technical.

A typical violet teaming exercise could span several weeks and involves the cross-functional roles outlined in Table 1. Teams begin by developing realistic scenarios, then conduct tabletop exercises as one would see where red team analysts probe technical vulnerabilities while blue team analysts monitor system responses. In parallel, human factors specialists assess workflow friction points, clinical champions validate usability under stress, and privacy officers evaluate compliance gaps. The system architect documents failure modes in real-time, and the policy liaison translates findings into actionable guidance.

**Table 1:** Development of a violet team for EHR cybersecurity practices. Common roles, responsibilities and key skills required. Adapted from (44).

Role	Responsibility	Key Skills
Red Team Analyst	Simulate adversarial attacks; expose technical and workflow vulnerabilities	In-Depth cybersecurity knowledge: Penetration testing, exploit development, threat modeling
Blue Team Analyst	Monitor systems; detect and respond to simulated and real threats	In-Depth cybersecurity knowledge; Security operations, intrusion detection, log analysis
Human Factors Specialist	Identify user friction, workflow stress, and trust issues in system interaction	Behavioral science, UI design
Clinical Champion	Represent frontline user concerns; validate system usability and sector relevance	Healthcare operations, clinical workflows, EHR experience
Privacy & Ethics Officer	Ensure patient data handling aligns with legal and ethical standards	Health law, data governance, patient consent, HIPAA/GDPR
System Architect/Engineer	Modify technical infrastructure to mitigate identified vulnerabilities	Systems integration, secure design principles, architecture review
Policy Liaison	Translate technical/user feedback into policy recommendations	Regulatory analysis, strategic communication

These exercises produce integrated deliverables, extending beyond those generated in a typical purple teaming exercise. Outputs include technical vulnerability reports, human factors assessments, and policy recommendations that explicitly connect technical findings to clinical and patient outcomes. Unlike traditional security assessments, violet teaming documents how vulnerabilities manifest in clinical practice, such as delays in accessing critical patient data or workflows that incentivize security bypass. This approach supports continuous adaptation as systems evolve, establishing an iterative cycle of assessment and improvement rather than one-time audits.

**Figure 1:**
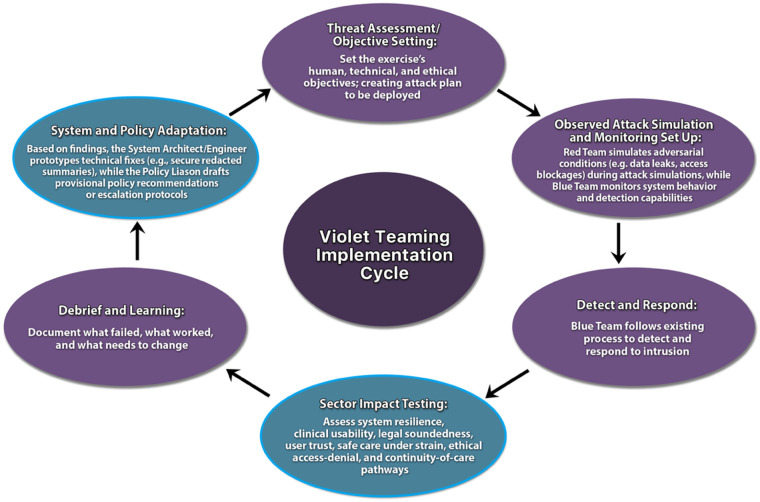
Violet teaming implementation cycle—understanding the violet teaming framework as a process. The Purple ovals represent actions typically found during normal red, blue, or purple teaming exercises. The blue ovals represent actions unique to the elements of Violet Teaming. The novelty of violet teaming relative to socio-technical frameworks is the explicit conversion of “values” into repeatable stress tests with defined remediation pathways (technical + policy), rather than standalone guidance or *post hoc* evaluation.

Figure 1 shows how violet teaming layers new steps onto purple teaming, expanding the cycle to test social impact and sector specific realities (45).

For EHRs, this means violet teaming tests more than data protection, it evaluates whether patients trust the process, whether clinicians can operate systems under stress, and whether policies match real-world practice. Instead of one-off exercises, violet teaming should become routine, with teams simulating scenarios to identify technical and human weaknesses.

In contrast to traditional approaches, red teams focus on identifying technical weaknesses, blue teams assess defensive readiness, and purple teams combine both to improve overall security posture. Violet teaming extends this by explicitly bridging technical risk with human-centered resilience, evaluating not only whether systems withstand attacks, but whether they remain usable, trustworthy, and ethically aligned under realistic conditions (1, 42).

To transition violet teaming to operational practice, we clearly define roles, responsibilities, and competencies for development. These roles include technical specialists, human-centered design experts, clinicians, and privacy officers. Violet teaming should recur across the lifecycle of EHR systems, with teams conducting collaborative simulations to test infrastructure while evaluating clinician workflows, user stress points, and patient trust outcomes. Such tabletop exercises can identify not just technical failures but also policy gaps, user misalignment, and sociotechnical vulnerabilities that cause patient/provider challenges. Table One outlines implementation roles for violet teaming within health information systems.

## The role of violet teaming in secure information exchange for military and civilian health data

### Vignette

A soldier was exposed to a chemical agent during a training accident. Initially, the exposure seemed minor, headaches and nausea passed quickly, and medical checks cleared him for duty. Years later, after discharge, he developed worsening health issues: fatigue, skin rashes, respiratory trouble, and neurological symptoms. When he sought care in the civilian system, critical information about his exposure was missing, his classified military health records were inaccessible to civilian providers. Without this context, doctors were forced to guess at the cause of his worsening conditions, resulting in worse care.

The scenario of the soldier exposed to a chemical agent during training demonstrates how conventional health information exchange systems creates gaps between military and civilian healthcare infrastructures, especially when information is classified. Although modern frameworks like FHIR were designed to make data sharing easier through standardized, API-based resources, many systems still lack consistent authentication and access controls. Legacy HL7 and DICOM pathways further compound these vulnerabilities. In practice, this means sensitive military exposure data may be withheld when urgently needed or, conversely, inadvertently exposed when protections differ across systems. As a result, patients like the soldier experience delays, misdiagnoses, and fragmented care.

These failures are worsened by inconsistent rules across systems. Military and civilian providers often operate under different definitions of sensitive data and incompatible access rights. There is no shared mechanism to determine when a combat-related injury should be restricted or when a civilian clinician should be granted essential context. Systems may block clinically relevant information or grant access without recognizing its sensitivity.

Violet teaming offers a corrective by focusing not only on technical hardening but on human outcomes and system usability. The red-team analyst would probe whether exposure data could be intercepted through weak endpoints, while a blue-team analyst would track cross-system data flows and flag anomalies such as civilian providers requesting records that never arrive. A comparable purple teaming exercise would stop at identifying these technical vulnerabilities and recommend improved authentication or encryption. Violet teaming extends past the technical evaluation by measuring downstream clinical and operational consequences. The exercise would track whether failed or blocked queries result in missing exposure data at the point of care, quantify delays in record retrieval, and assess whether clinicians receive incomplete summaries. Potential outcomes would include the percentage of exposure records successfully retrieved across systems, time-to-access for critical data elements, frequency of access denials due to mismatched classification rules, and clinician interpretation accuracy when exposure data is partially redacted. A human-factors assessment would document whether interface-level signals clearly communicate why data is unavailable, while a clinical reviewer would evaluate whether substituted summaries preserve patient utility. The resulting violet-team outputs therefore differ from purple-team findings by linking technical failures to clinical impact and decision quality, ultimately producing recommendations not only for endpoint security, but for redesigning access logic and policy thresholds when sensitive military health information can be shared securely and effectively across systems.

## Discussion

Interoperability between military and civilian EHRs remains a stubborn vulnerability, especially when patients transition between systems. Despite investments in data standards and compliance frameworks, failures persist, not because core functions are missing, but because systems are rarely designed or tested for the full complexity of real clinical workflows. Service members and veterans often face delays, incomplete records, or misinterpretation when medical histories cross networks with incompatible standards. Too often, sensitive data is either inaccessible when needed or shared without proper oversight.

These breakdowns rarely stem from external cyber threats alone; they arise from the internal limitations of EHR ecosystems testing or poor tagging, outdated APIs, or credential mismatches. Red, blue, and purple teaming focus on technical defenses but overlook whether those defenses align with practical workflows. As a result, critical failures go unnoticed until they harm patients.

Violet teaming closes this gap. It embeds scenario-based tests that combine technical simulations with operational and ethical checks throughout the entire lifecycle of the system, from design and training through development and sustainment. Unlike traditional approaches, violet teaming blends red and blue teams with privacy officers, human factors experts, and clinicians. This multidisciplinary approach precludes misalignment between workflows, governance, or user behaviors. It supports ongoing, adaptive tests as systems evolve. This is critical as AI becomes more embedded in EHRs and may risk reliance on incomplete or biased data in veteran health contexts.

Violet teaming can also reveal the gaps between legacy and modern standards. Many military or rural sites still rely on older HL7 frameworks, while others adopt FHIR. On paper, they're interoperable, but in practice inconsistencies can break connections. Violet teaming stress-tests these bridges, pinpointing failures that standard compliance checks miss.

Most importantly, it tests not just whether data is secure, but whether patients and providers can use it when it matters most. Lightweight violet teaming cycles can target high-risk handoffs, between DoD and VA, or TRICARE, and civilian hospitals, without full audits. Over time, these reveal vulnerabilities, guiding updates to protocols, infrastructure, and training.

In addition, the emergence of national-scale governance efforts such as the TEFCA reflects like efforts at the policy level. TEFCA's “network of networks” model establishes a common legal and technical baseline for data exchange across disparate systems. However, while TEFCA advances structural connectivity, it does not fully address whether exchanged data is usable or interpretable within the contexts in which care is delivered, thus compliance with exchange frameworks does not guarantee operational effectiveness.

Violet teaming complements TEFCA by operating within and across these governance structures to test how interoperability performs under realistic conditions. Rather than replacing regulatory frameworks, it functions as validation, stress-testing qualified health information networks (QHIN), through scenario-based simulations that reflect clinical and technical complexity. For example, violet teaming can evaluate whether a service member's exposure history, transmitted through a TEFCA-aligned network, is correctly interpreted by a civilian provider, appropriately filtered based on sensitivity, and available at the point of care. In doing so, it bridges the gap between policy-level interoperability and frontline usability.

Violet teaming remains an emerging concept, with only a limited number of publications describing its principles and potential benefits. Accordingly, this piece should be viewed as a foundational contribution rather than evaluation, as this work aims to clarify the method, outline initial operational strategies with a prominent example, and provide a basis for future empirical testing and refinement by researchers and practitioners as cyber practices continue to evolve with AI.

In short, violet teaming tackles what conventional security often misses: failures of coordination, context, and real-world fit. By simulating how people, policies, and systems interact throughout the entire system development cycle, it reveals insights for effective care and opportunities to strengthen resilience, especially when military and civilian health networks intersect.

## Data Availability

The original contributions presented in the study are included in the article/Supplementary Material, further inquiries can be directed to the corresponding author.
